# Bloodstream Infections Caused by *Klebsiella pneumoniae* Carbapenemase–Producing *P. aeruginosa* Sequence Type 463, Associated With High Mortality Rates in China: A Retrospective Cohort Study

**DOI:** 10.3389/fcimb.2021.756782

**Published:** 2021-11-01

**Authors:** Hangbin Hu, Yan Zhang, Piaopiao Zhang, Jie Wang, Qing Yuan, Weixiao Shi, Sheng Zhang, Haiting Feng, Yunbo Chen, Meihong Yu, Hongchao Chen, Yan Jiang, Qing Yang, Tingting Qu

**Affiliations:** ^1^ State Key Laboratory for Diagnosis and Treatment of Infectious Diseases, Collaborative Innovation Center for Diagnosis and Treatment of Infectious Disease, National Clinical Research Center for Infectious Diseases, Zhejiang University School of Medicine First Affiliated Hospital, Hangzhou, China; ^2^ Respiratory Department, The First Affiliated Hospital, Zhejiang University School of Medicine, Hangzhou, China; ^3^ Infection Control Department, The First Affiliated Hospital, Zhejiang University School of Medicine, Hangzhou, China; ^4^ Department of Laboratory Medicine, The First Affiliated Hospital, Zhejiang University School of Medicine, Hangzhou, China; ^5^ Department of Infectious Diseases, Sir Run Run Shaw Hospital, Zhejiang University School of Medicine, Hangzhou, China

**Keywords:** bloodstream infections, hypervirulence, carbapenem-resistant *Pseudomonas aeruginosa*, KPC, ST463

## Abstract

**Objectives:**

Recently, KPC-producing *P. aeruginosa* has rapidly emerged and expanded in East China. Here we described the clinical impact and characteristics of bloodstream infections (BSIs) from the dominant KPC-producing CRPA belonging to Sequence Type (ST) 463.

**Methods:**

Retrospective cohort study was performed with CRPA BSI cases from 2019 to 2020 in a hospital in East China. Clinical characteristics, risk factors, and all-course mortality were evaluated. All CRPA isolates had whole-genome sequencing, antimicrobial susceptibility testing, and serum resistance assay. Representative isolates were tested for virulence in a *Galleria mellonella* infection model.

**Results:**

Among the 50 CRPA BSI cases, ST463 predominated (48.0%). In multivariate analysis, we found three independent risk factors for fatal outcome: KPC carriage (OR 4.8; CI95% 1.0-23.7; *P =* 0.05), Pitt bacteremia score (OR 1.3; CI95% 1.0-1.6; *P =* 0.02), and underlying hematological disease (OR 8.5; CI95% 1.6-46.4; *P =* 0.01). The baseline clinical variables were not statistically different across STs, however the 28-day mortality was significantly higher in ST463 cases than that in non-ST463 cases (66.7% *vs* 33.3%, *P* = 0.03). *ExoU* and *exoS* virulence genes coexisted in all ST463 isolates, and the carbapenem resistant gene *bla*_KPC_ were produced in almost all ST463 isolates, significantly higher than in the non-ST463 group(95.8% *vs* 7.7%, P<0.001). ST463 CRPA isolates also showed higher resistance rates to antipseudomonal cephalosporins, monobactam, and fluoroquinolones. And ST463 CRPA was confirmed hypervirulence in the larvae model. The genome of one ST463 CRPA strain showed that the *bla*_KPC-2_ gene was the sole resistance gene located on a 41,104bp plasmid pZYPA01, carried on a 7-kb composite transposon-like element flanked by two IS*26* elements (IS*26*–Tn*3*-tnp*A*–IS*Kpn27*–*bla*_KPC-2_–IS*Kpn6*–IS*26*). Plasmid from various species presented core *bla*_KPC-2_ was franked by mobile genetic element IS*Kpn27* and IS*Kpn6.*

**Conclusions:**

In the ST463 CRPA BSI cohort, the mortality rates were higher than those in the non-ST463 CRPA BSI. The ST463 CRPA clone coharboring the *bla*_KPC_ and *exoU/exoS* genes emerged and spread in East China, which might develop to a new threat in the clinic. Our results suggest that the surveillance of the new high-risk clone, ST463 CRPA, should be strengthened in China, even worldwide in the future.

## Introduction

*Pseudomonas aeruginosa* (PA) is a common and serious cause of nosocomial bloodstream infection; it is often severe when patients have compromised immunity. Despite improvements in medical care, the mortality of PA bacteremia remains considerably high (Thaden et al., 2017). Mortality in PA bloodstream infection is multifactorial, including the immune status of the host, the initial site of infection, the antibiotic treatment, and the microorganism (Hu et al., 2021a). Moreover, the presence of carbapenem-resistant *Pseudomonas aeruginosa* (CRPA) limits the treatment options and increases the risk of inadequate empirical therapy (Doi, 2019).

The common resistance mechanisms of *P. aeruginosa* to carbapenems are the loss of the outer membrane protein OprD and the overexpression of efflux pumps and/or the intrinsic chromosomally encoded AmpC *β*-lactamase (Li et al., 2012; Potron et al., 2015; Pang et al., 2019). Another mechanism for carbapenem resistance is the production of carbapenemases such as Metallo-*β*-lactamases. KPC-producing (class A *β*-lactamase) *P. aeruginosa* isolates have been occasionally reported in some countries since 2006 (Naas et al., 2013; Hu et al., 2015; Hagemann et al., 2018; Walkty et al., 2019). The *bla*_KPC_ genes were frequently detected globally mainly among the species in the family *Enterobacteriaceae* (Feng et al., 2017). Some studies reported that *bla*_KPC_ in CRPA might be transmitted from carbapenem-resistant *Enterobacteriaceae*. In China, KPC-2-producing *Pseudomonas aeruginosa* ST463 was first reported in 2015 and has rapidly spread in Zhejiang in recent years (Hu et al., 2015). However, clinical studies of BSIs by the emerging ST463 *P. aeruginosa* are still lacking.

In PA strains, the type III secretion system (T3SS) play a major role in their intrinsic virulence levels (Hauser, 2009). This secretion system injects potent cytotoxins into the cell and includes four effector proteins: *exoU*, a phospholipase; *ExoS* and *ExoT*, bifunctional proteins, and ExoY, an adenylate cyclase (Engel and Balachandran, 2009). In recent years, the cytotoxic *exoU* virulence gene of *P. aeruginosa* has been found to be associated with severe disease and poor outcomes. Furthermore, it is an important independent marker of early death in patients with *P. aeruginosa* BSI (Halavaty et al., 2012; Sawa et al., 2014; Peña et al., 2015). Almost all strains encode for *exoT* and *exoY* (Feltman et al., 2001), but the coexistence of *exoU* and *exoS* genes has rarely been reported due to their frequent mutual exclusivity (Hauser, 2009; Bradbury et al., 2010; Oliver et al., 2015). Previous studies indicated that the *exoU* genotype was also related to individual resistance to several antipseudomonal agents, including fluoroquinolones, carbapenems, and cephalosporins (Engel and Balachandran, 2009; Peña et al., 2015). However, one report illustrated that the co-presence of *exoU* and *exoS* was associated with a greater capacity for multi-drug resistance (Horna et al., 2019).

Herein, we described the occurrence of BSIs caused by KPC-producing CRPA that belonged to ST463. We investigate the high mortality rates associated with it in a tertiary hospital in East China in 2019–2020. We characterized the genomic alterations in the ST463 CRPA population and ascertained associated changes in phenotype and pathogenicity traits.

## Materials And Methods

### Study Population

**Figure 1:**
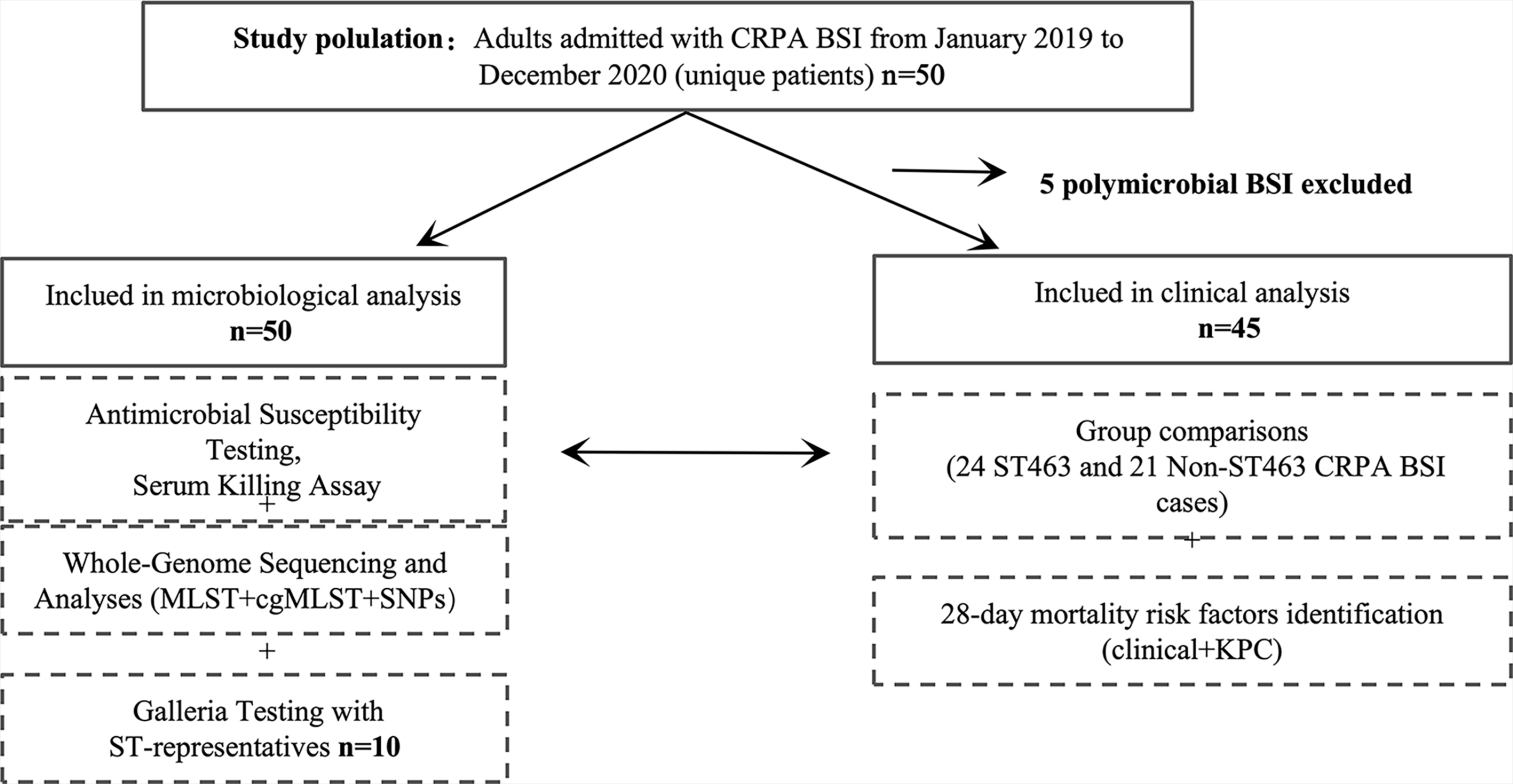
Study design. BSI, bloodstream infection; MLST, mulitilocus sequence typing; cgMLST, core genome MLST; SNPs, single nucleotide polymorphisms.

We conducted a retrospective cohort study of CRPA BSI cases in a 2,500-bed public teaching hospital located in Zhejiang, China. The cases of CRPA BSIs were retrieved from the conventional microbiology laboratory database. The hospitalized patients with positive CRPA blood culture from January 2019 to December 2020 were selected. Five polymicrobial BSI cases were excluded from clinical analysis, but not from microbiological analysis. The study flowchart is shown in **Figure 1**. Only the first isolate was selected for each patient. This study was approved by the institutional review board of the First Affiliated Hospital of Zhejiang University in China (approval no. IIT20210120B). Informed consent was waived by the review board due to the nature of the study. A standardized case record form was used to collect epidemiological and clinical data, as detailed in the Supplementary Materials.

### Microbiological Analysis

Frozen CRPA isolates were cultured and reidentified by VITEK 2 system (bioMérieux, France). We confirmed minimum inhibitory concentrations (MICs) of twelve antipseudomonal agents. Imipenem, meropenem, piperacillin-tazobactam, ceftazidime, cefepime, amikacin, gentamicin, ciprofloxacin, levofloxacin, aztreonam, and ceftazidime-avibactam were determined by the agar dilution method. The broth micro-dilution method was used to quantify antibacterial resistance against polymyxin. The results were interpreted according to Clinical and Laboratory Standards Institute (CLSI, 2021) standards (Papp-Wallace et al., 2010). All 50 CRPA isolates were sequenced using an Illumina Hiseq 2500 instrument (Qiagen, Valencia, CA, USA), and annotation was provided using the RAST server. Multi-locus sequence typing (MLST) and antibiotic resistance genes were conducted by the CGE server (https://cge.cbs.dtu.dk). Virulence factors were searched using the Virulence Factor DataBase (http://www.mgc.ac.cn/VFs/) (Liu et al., 2019). To determine the clonal relatedness, we performed a genome-wide gene-by-gene comparison by applying the SeqSphere+ software. Single nucleotide polymorphisms (SNPs) analysis was further conducted on CRPA isolates of the same Sequence Type clone. One isolate (ZYPA01) was carried out with long-read sequencing using the PacBio RSII platform. Sequence identity among the *bla*_KPC-2_ flanking genes of the isolate and KPC-encoding plasmids from *P. aeruginosa* reported previously, *Escherichia coli* or *K. pneumoniae* isolates were assessed by Blast analysis. Descriptions of detailed bioinformatics analysis are available in the Supplementary material.

### Virulence Studies

We did a serum resistance assay with all 50 CRPA strains. We randomly selected five ST463 CRPA (ZYPA06, ZYPA10, ZYPA29, ZYPA32, and ZYPA36) and five non-ST463 CRPA (ZYPA08, ZYPA11, ZYPA23, ZYPA27, and ZYPA41) isolates as the representative for *G. mellonella* infection tests. The two assays were modified according to previously described protocols and are described in the **Supplementary Methods**. *P. aeruginosa* PAO1 was used as control.

### Statistical Analysis

χ2 or Fisher exact test was used for categorical variables and t-test or Mann-Whitney U test for continuous variables to compare different groups in our study. Univariate analysis and multivariate analysis were performed to determine the risk factors for 28-day mortality. Variables with a *P* value of ≤ 0.05 in the univariate analysis were used in the binary logistic regression analysis to identify independent predictors. Kaplan-Meier survival curves (log-rank test) were obtained with the survminer R package. Kaplan-Meier survival curves were obtained in different groups. Analyses were two-tailed and the P-value of < 0.05 was considered significant. SPSS for Windows (IBM^®^, version 16.0) was used for the analysis.

## Results

### Overall Cohort Clinical Data

In this two-year analysis, we extracted and analyzed clinical data from the database for 45 patients with CRPA BSIs except for five polymicrobial cases. The epidemiological characteristics, clinical features, treatments, and clinical outcomes of the patients are listed in **Table 1**. The median age of our cohort was 56 years and 37.8% were over 65 years. Most of the patients were male (71.1%). Majority of the patients had hematologic conditions (19, 42.2%). More than half (55.7%) of the patients had a prior hospitalization, with a median length of stay of 19 days, and 35.6% had been admitted to the intensive care unit. Most patients had invasive procedures prior to CRPA BSI: 66.7% of the patients underwent central venous catheterization, followed by mechanical ventilation (47.7%) and percutaneous catheterization (42.2%). Bacteremia was predominantly from respiratory tract infections (28.9%), followed by catheter-related and urinary tract infections (both 8.9%). Inflammatory indicators were elevated when bacteremia occurred. The median white blood cell (WBC) was 7.3*10^9^/L, C-reactive protein (CRP) was 105.5 g/mL (increased), and procalcitonin (PCT) was 2.6 pg/mL (increased). Carbapenems combined with aminoglycosides were the most common antibiotic treatment (n = 15, 33.3%), followed by carbapenems combined with polymyxin B (n = 14, 31.1%). All-cause mortality rate at 7, 14 and 28 days was 44.4%, 48.9% and 51.1%, respectively. The clinical description of the 24 ST463 CRPA BSI cases is shown in **Supplementary Table S1**.

**Table 1:** Characteristics of patients with bloodstream infections caused by Carbapenem-Resistant *P. aeruginosa* according to sequence typing.

Characteristic	Total (n = 45)	ST463 (n = 24)	Non-ST463 (n = 21)	*P* value
Demographic				
Sex (male)	32 (71.11)	18 (75.00)	14 (66.67)	0.54
Age, years, median (IQR)	56 (32.00-71.00)	46.5 (25.25-66.00)	65 (33.50-76.50)	0.06
Elderly (age>65y)	17 (37.78)	7 (29.17)	10 (47.62)	0.20
BMI, kg/m2, mean (SD)	21.71 (3.02)	21.55 (2.30)	21.97 (4.05)	0.74
Underlying medical conditions				
Diabetes	6 (13.33)	2 (8.33)	4 (19.05)	0.54
Lung disease	3 (6.67)	2 (8.33)	1 (4.76)	1.00
Kidney disease	5 (11.11)	2 (8.33)	3 (14.29)	0.87
Hepatic disease	8 (17.78)	4 (16.67)	4 (19.05)	1.00
Benign biliary diseases	2 (4.44)	0 (0)	2 (9.52)	0.21
Cardiovascular diseases	15 (33.33)	6 (25.00)	9 (42.86)	0.20
Hematological disease	19 (42.22)	12 (50.00)	7 (33.33)	0.26
Solid malignant tumor	3 (6.67)	1 (4.17)	2 (9.52)	0.90
Solid-organ transplant	6 (13.33)	4 (16.67)	2 (9.52)	0.79
Hospital stay before BSI, median (IQR)	19 (9.50-30.50)	20 (9.25-36.50)	18 (10.00-28.50)	0.36
Nosocomial	38 (84.44)	22 (91.67)	16 (76.19)	0.31
Trauma	3 (6.67)	2 (8.33)	1 (4.76)	1.00
Surgery	22 (48.89)	12 (50.00)	10 (47.62)	0.87
Pitt bacteremia score, median (IQR)^a^	3 (1.00-7.00)	3.5 (1.00-9.25)	2 (0.50-6.50)	0.31
Prior hospitalization (2 weeks)	25 (55.56)	13 (61.90)	12 (50.00)	0.42
ICU (prior to bacteremia onset)	16 (35.56)	9 (37.50)	7 (33.33)	0.77
CRPA BSI prior to hemodialysis	11 (24.44)	7 (29.17)	4 (19.05)	0.43
Prior invasive procedure and/or devices				
Mechanical ventilation	21 (47.73)	12 (50.00)	9 (45.00)	0.74
Urinary catheterization	12 (27.91)	7 (29.17)	5 (26.32)	0.84
Central venous catheterization	30 (66.67)	16 (66.67)	14 (66.67)	1.00
Percutaneous catheterization	19 (42.22)	13 (54.17)	6 (28.57)	0.08
Laboratory examination				
White blood cell, median (IQR)^b^	7.2 (0.33-15.30)	2.9 (0.23-11.85)	12.5 (0.70-15.60)	0.16
Neutrophilic granulocyte, median (IQR)c	5.6 (0.06-13.30)	1.8 (<0.01-10.40)	10.9 (0.20-14.10)	0.21
Red blood cell, median (IQR)^b^	2.31 (2.03-3.41)	2.21 (1.94-2.66)	2.39 (2.07-3.88)	0.14
Hemoglobin, median (IQR)^b^	69 (62.50-105.50)	67.5 (61.25-86)	71 (63-117)	0.23
Platelet, median (IQR) a	50 (11.00-247.50)	20 (9-168)	118 (25-252)	**0.04**
C-reactive protein, median (IQR)^c^	105.50 (61.60-158.94)	119.75 (84.67-160.17)	86.9 (42.60-160.14)	0.23
Procalcitonin, median (IQR)^c^	2.56 (0.42-15.05)	2.68 (0.57-15.57)	1.86 (0.21-17.67)	0.51
Albumin, median (IQR)^d^	27.90 (24.30-29.93)	27.10 (20.80-28.40)	29.30 (25.40-31.90)	**0.03**
Agranulocytosis	18 (40.00)	11 (45.83)	7 (33.33)	0.39
Source of bacteremia				
Lung	13 (28.89)	5 (20.83)	8 (38.10)	0.20
Intra-abdominal infection^e^	2 (4.44)	1 (4.17)	1 (4.76)	1.00
Skin and soft-tissue infection	3 (6.67)	2 (8.33)	1 (4.76)	1.00
Surgical sites	1 (2.22)	1 (4.17)	0 (0.00)	1.00
Catheter related	4 (8.89)	1 (4.17)	3 (12.50)	0.70
Urinary tract infection	4 (8.89)	3 (12.50)	1 (4.76)	0.70
Unknown	19 (42.22)	10 (41.67)	9 (42.86)	0.94
Treatment				
Hormonal therapy	29 (64.44)	17 (70.83)	12 (57.14)	0.34
Other immunotherapy	20 (44.44)	12 (50.00)	8 (38.10)	0.42
Antibiotic After BSI				
Carbapenems + aminoglycosides	15 (33.33)	5 (20.83)	10 (47.62)	0.06
Carbapenems + polymyxin B	14 (31.11)	4 (16.67)	10 (47.62)	**0.03**
Ceftazidime-avibactam + aminoglycosides	7 (15.56)	1 (4.17)	6 (28.57)	0.07
Ceftazidime-avibactam + carbapenems	1 (2.22)	0 (0.00)	1 (4.76)	0.47
Ceftazidime-avibactam + polymyxin B	4 (8.89)	3 (12.50)	1 (4.76)	0.70
Mortality				
All-cause death at 7 d postbacteremia	20 (44.44)	14 (58.33)	6 (28.57)	0.05
All-cause death at 14 d postbacteremia	22 (48.89)	15 (62.50)	7 (33.33)	0.05
All-cause death at 28 d postbacteremia	23 (51.11)	16 (66.67)	7 (33.33)	**0.03**

Data are presented as no. (%) unless otherwise indicated. Bolded numbers indicate that P < 0.05.

IQR, interquartile range; BMI, body mass index; SD, standard deviation; IAI, intra-abdominal infection; ICU, intensive care unit; BSI, bloodstream infection.

a. Evaluated 48 hours before or 24 hours after the first positive blood culture, whichever is the highest.

b. CRPA detected in the first blood culture.

c. The highest value before and after CRPA48 was detected in blood culture.

d. Lowest value at onset.

e. Including biliary tract and enteric canal.

### Clinical Analysis According to Sequence Type

**Figure 2:**
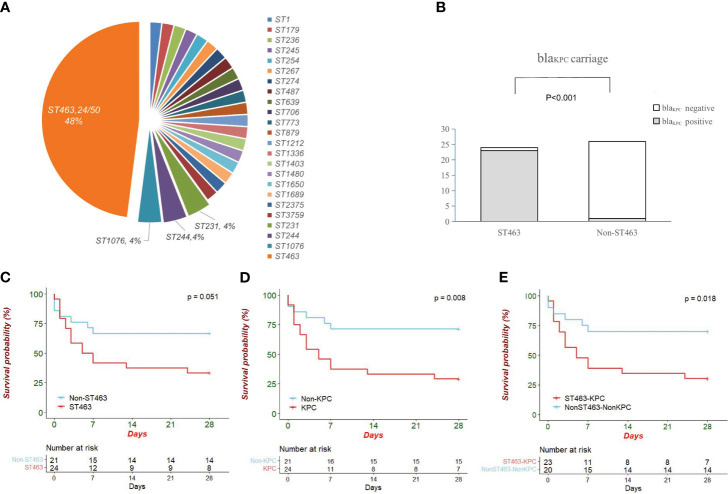
**(A)** Pie chart showing sequence type distribution of 50 CRPA isolates; **(B)** Comparison of *bla*_KPC_ carriage between ST463 and Non-ST463 strains (Fisher’s Exact Test). ST463 and *bla*_KPC_ are highly associated (23 out of 24; 95.8%); **(C)** The Kaplan–Meier survival curves in patients infected with ST463 CRPA and with non-ST463 CRPA (*P* = 0.05); **(D)** The Kaplan–Meier survival curves between the two groups of patients infected with KPC-producing CRPA and non-KPC-producing CRPA (*P* = 0.008); **(E)** The K–M survival curves between KPC-producing ST463 CRPA and other STs CRPA that do not carry *bla*_KPC_ (P = 0.018). The *P* value of Kaplan Meier’ analysis was calculated with log-rank test.

The 50 CRPA strains isolated from patients belonged to 24 different STs. As shown in **Figure 2A** (MLST plot), ST463 was the predominant type (48.0%, 24/50), and the other 26 strains showed a high degree of ST diversity. The rate of the *bla*_KPC_ gene in ST463 CRPA was significantly higher than in non-ST463 CRPA (23/24, 95.8% *vs* 2/26, 6.7%; *P* < 0.001, **Figure 2B**).

After the exclusion of five polymicrobial BSIs cases, patients were divided into two groups, ST463 CRPA-infected (n=24) and non-ST463 CRPA-infected (n=21), to determine whether there was specificity in the clinical features of ST463-infected patients. Notably, all-cause mortality at days 7, 14, and 28 was higher in ST463-infected than in non-ST463-infected patients (58.3% *vs* 28.6%, 62.5% *vs* 33.3%, and 66.7% *vs* 33.3%, respectively) (**Table 1**). The 28-day survival curves based on different STs also showed a significant difference (*P* = 0.03) as detailed in **Figure 2C**. Furthermore, the ST463-infected group had a higher median Pitt bacteremia score of 3.5 compared with the non-ST463-infected group with a score of 2 (*P =* 0.3); this score is a predictor of early mortality risk in patients with BSI. There were no significant differences found in the clinical variables such as age, gender, underlying disease, or treatment received between the two groups; however, the amount of platelets was lower in the ST463-infected group (20 *vs* 118 *109/L, P=0.04) (**Table 2**).

**Table 2:** Analysis of risk factors for 28-Day mortality in 45 patients with Carbapenem-Resistant *P. aeruginosa* bloodstream infection.

Covariate	Univariate Analysis			Multivariate Analysis	
	Survived (n = 22)	Died (n = 23)	*P* value	OR (95% CI)	*P* value
Sex (male)	14 (63.64)	18 (78.26)	0.28		
Age, years, mean (SD)	54.86 (23.66)	48 (22.54)	0.32		
*bla*_KPC_	7 (31.82)	17 (73.91)	**<0.01**	4.87 (1.00-23.68)	**0.05**
Underlying medical conditions					
Hematological disease	4 (18.18)	15 (65.22)	**<0.01**	8.53 (1.57-46.35)	**0.01**
Solid malignant tumor	2 (9.09)	1 (4.35)	0.97		
Solid-organ transplant	1 (4.55)	4 (17.39)	0.37		
Pitt bacteremia score, median (IQR)^a^	1.5 (0-3.25)	6 (2-12)	**0.01**	1.3 (1.04-1.64)	**0.02**
Source of bacteremia					
Respiratory tract infection	7 (31.82)	7 (30.43)	0.92		
Intra-abdominal infection^b^	1 (4.55)	1 (4.35)	1		
Skin and soft-tissue infection	0 (0)	3 (13.04)	0.25		
Surgical sites	1 (4.55)	0 (0)	0.49		
Catheter related	1 (4.55)	1 (4.35)	1		
Urinary tract infection	3 (13.64)	1 (4.35)	0.57		
Unknown	9 (40.91)	10 (43.48)	0.86		
Treatment^c^					
Carbapenems + aminoglycosides	12 (54.55)	11 (47.83)	0.65		
Carbapenems + polymyxin B	14 (63.64)	13 (56.52)	0.63		
Ceftazidime-avibactam + aminoglycosides	9 (40.91)	6 (26.09)	0.29		
Ceftazidime-avibactam + carbapenems	1 (4.55)	0 (0)	0.49		
Ceftazidime-avibactam + polymyxin B	2 (9.09)	2 (8.70)	1		

OR, odds ratio; 95%CI, 95% confidence intervals; SD, standard deviation; IQR, inter-quartile range. Bolded p-values indicate P ≤ 0.05.

Variables showing P value ≤ 0.1 in the univariate analysis were further included in the multivariate model (binary logistic regression);

a Evaluated 48 hours before or 24 hours after the first positive blood culture, whichever is the highest;

b Including the biliary tract and enteric canal;

c Antibiotic treatment after BSI.

### Mortality Predictor Analysis

Factors that may affect 28-day mortality in this CRPA BSI cohort were described in **Table 2**. Multivariate analysis confirmed the following as independent factors: *bla*_KPC_ carriage (odds ratio [OR], 4.9; 95% confidence interval [CI], 1.0-23.7; *P =* 0.05), Pitt bacteremia score (odds ratio [OR], 1.3; 95% confidence interval [CI], 1.0-1.6; *P =* 0.02), and underling hematological disease (odds ratio ([OR], 8.5; 95% confidence interval [CI], 1.6-46.4; *P =* 0.01).

### Isolates’ Genetic Relatedness, Antimicrobial Susceptibility Testing, Antibiotic Resistance Genes, and Virulence Factors

**Figure 3:**
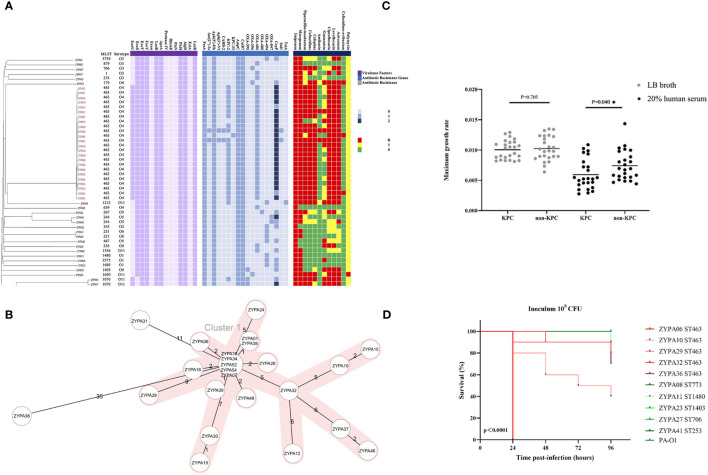
**(A)** Genetic relatedness, antibiotic resistance genes, virulence factor determinants, and antimicrobial susceptibility testing results of 50 CRPA BSI strains. These features are shown separately on the four blocks of the picture. The gray value indicates the copy number of the antibiotic resistance gene and virulence factors *bla*_KPC-2_ and ST463 are highly associated (22 out of 24; 91.7%). R, resistant; I, Intermediate; S, Sensitive; **(B)** Minimum spanning trees of 24 ST463 CRPA BSI strains from cgMLST analysis. Distance based on 4823 targets genes with pairwise ignore missing values. CgMLST profiles are represented by circles. The shaded parts represent the same cluster with a distance threshold of 10. The size of the circle is proportional to the number of isolates sharing the same cgMLST profile, with the biggest one including five isolates; **(C)** Neutrophil-killing resistance; at the maximum growth rate of KPC and non-KPC group; **(D)** Virulence potential in a *G mellonella* infection model. Five isolates of ST463 and five isolates of other STs representing various genetic background were randomly selected for the infection assay at 10^5^ CFU. *P* values were calculated by log-rank (Mantel-Cox) test. Each line represents a single isolate. Isolate PA-O1 was used as the control. Abbreviation: CFU, colony-forming units.

MLST and serotype analysis revealed that the 50 clinical strains belonged to 24 MLST types and eight serotypes, and *P. aeruginosa* ST463 strains were associated with serotype O4. The connections between serotypes and STs in other isolates are shown in **Figure 3A**. The same figure also depicts the antibiotic resistance genes identified in the CRPA genome. In our study, *bla*_KPC_ was found in the genomes of 23/24 ST463 isolates, including 22 *bla*_KPC-2_ and 1 *bla*_KPC-33_; on the other hand, it was found in only one non-ST463 isolate (95.8% *vs* 3.8%, *P* < 0.001). The prevalence of the fluoroquinolone-resistance gene (*crpP*) was also significantly higher in the ST463 group than in the non-ST463 group, and most ST463 isolates had two copies of the *crpP* gene (**Figure 2A**). All ST463 strains carried *bla*_OXA-486_ *β*-lactamase gene, while the non-ST463 strains were genotyped with dispersed oxacillinases. Other resistance genes did not show differences between the two groups. We also found some significant differences in T3SS virulence factors between ST463 and non-ST463 CRPA strains (**Figure 2A**). All ST463 isolates coharbored *exoU* and *exoS* genes. However only 4/26 (15.4%) of the non-ST463 CRPA isolates carried *exoU* gene, and the other 22/26 non-ST463 strains carried the e*xoS* gene without *exoU*.

Antimicrobial susceptibilities were summarized in **Table 3** and **Figure 2A**. ST463 CRPA isolates were highly resistant to imipenem and meropenem (MIC_50_, 256 mg/L; 100% resistance). The MIC_50_s of carbapenems for ST463 CRPA isolates were 8- to 16-fold higher than for non-ST463 CRPA isolates: imipenem (MIC_50_, 32 mg/L; 100% resistance) and meropenem (MIC_50_, 16 mg/L; 84.6% resistance). Also, ST463 isolates show statistically significant difference in higher resistant rates to piperacillin-tazobactam (91.7% *vs* 26.9% resistance), ciprofloxacin (100% *vs* 38.5% resistance), levofloxacin (100% *vs* 42.3% resistance), ceftazidime (95.8% *vs* 19.2% resistance), cefepime (95.8% *vs* 30.8% resistance), and aztreonam (100% *vs* 42.3% resistance), to those of non-ST463 CRPA isolates (*P* < 0.001). Notably, all of the CRPA were less frequently resistant to ceftazidime-avibactam, amikacin and polymyxin.

**Table 3:** Antimicrobial susceptibilities of 24 ST463 and 26 Non-ST463 Carbapenem-Resistant *P. aeruginosa* isolates.

Antibiotic(s)	ST463 (n = 24)				Non-ST463 (n = 26)				*P* value (R%)
	Resistance rate (%)	MIC50 (μg/mL)	MIC90 (μg/mL)	MIC range (μg/mL)	Resistance rate (%)	MIC50 (μg/mL)	MIC90 (μg/mL)	MIC range (μg/mL)	
Imipenem	100	256	256	32 - 512	100	32	256	8 - 256	1
Meropenem	100	256	256	128 - 512	84.6	16	256	0.125 - 256	0.138
Piperacillin-tazobactam	91.7	>256	>256	16 - 512	26.9	16	256	4 - 512	**<0.001**
Ceftazidime	95.8	256	256	8 - 256	19.2	8	256	2 - 128	**<0.001**
Cefepime	95.8	128	256	16 - 512	30.8	8	128	2 - 256	**<0.001**
Amikacin	12.5	8	256	2 - 256	0	8	16	1 - 32	0.206
Gentamicin	20.8	8	256	2 - 256	30.8	8	16	1 - 32	0.424
Ciprofloxacin	100	32	64	16 - 64	38.5	1	32	0.25 - 32	**<0.001**
Levofloxacin	100	128	128	32 - 128	42.3	4	64	1 - 128	**<0.001**
Aztreonam	100	256	256	32 - 512	42.3	16	256	1 - 256	**<0.001**
Ceftazidime-avibactam	8.3	8	8	2 - 256	3.8	2	8	1 - 32	0.943
Polymyxin	0	1	1	1 - 2	3.8	1	2	1 - 4	1

Bolded p-values indicate P ≤ 0.05.

Based on sequence analysis of the whole genome, the phylogenetic tree showed that 22 of the 24 ST463 CRPA isolates belonged to the same cgMLST cluster 1, except for isolates of ZYPA36 and ZYPA31 (**Figure 3B**). For further core-genome SNPs, the average pairwise distance between two isolates is 99.95 SNPs and ranges from 1 to 2273 SNPs. Within the ST463 group, one strain genome (ZYPA01) was fully assembled for further analysis. pZYPA01, a unique plasmid, carried the sole resistance gene *bla*_KPC-2,_ and no virulence genes were found (**Supplementary Figure S1A**). It could not be assigned to any known incompatibility group. Blast analysis of the pZYPA01 sequence revealed that it was highly similar to pPA1011 (GenBank accession number MH734334.1), a plasmid carrying carbapenem-resistant gene *bla*_KPC-2_ from an ST463 *P. aeruginosa* isolate in Hangzhou, China, with 99.1% identities and 99.0% query coverage (**Supplementary Figure S1A**). The plasmid pZYPA01 sequence was also highly similar to the plasmid sequence harboring the *bla*_KPC-33_ (pZYPA54), with 99.1% identities and 99.0% query coverage. The core *bla*_KPC-2_ platform Tn*3*-tnp*A*–IS*Kpn27*–*bla*_KPC-2_–IS*Kpn6* had also been found in pPA1011 from *P. aeruginosa*, pKP048 from *K. pneumoniae* and pHS102707 from *Escherichia coli* (**Supplementary Figure S1B**). Unlike the mobile genetic element described in pPA1011, pKP048, and pHS102707 (Tn*3*–IS*Kpn8*–*bla*_KPC-2_-IS*Kpn6*), *bla*_KPC-2_ in pZYPA01 was carried on a 7-kb composite transposon-like element flanked by two IS*26* elements (IS*26*–Tn*3*-tnp*A*–IS*Kpn27*–*bla*_KPC-2_–IS*Kpn6*–IS*26*), hence making it potentially transferable.

### Virulence Assessment With Serum Killing and *G. mellonella* Infection Assays

In our study, all strains were tested in 20% human serum to investigate their capacity to resist the serum bactericidal activity. Subgroup analysis showed that the maximum growth rate of KPC-positive isolates was significantly lower compared to -negative isolates (**Figure 3C**, *P =* 0.04). Although there was no statistical difference, the maximum growth rate was also lower in the ST463-CRPA group than in the non-ST463-CRPA (*P =* 0.24).

We randomly selected five representative ST463 isolates and five non-ST463 isolates to test the virulence properties of these strains. Continuous monitoring for 96 hours, even at the lowest concentration (10^5^ cfu), the mortality rate of the ST463-infected larvae group was higher than in the non-ST463-infected. Of all isolates tested, the ST463 isolates PAZY06 were the most virulent strains in all inocula tested (**Figure 3D**).

## Discussion

A recent increase in the detection rate of CRPA has been observed in Zhejiang Province, China, from 22% in 2015 to 32% in 2020; it is also significantly higher than in other provinces (Hu et al., 2019; Hu et al., 2021a; Hu et al., 2021b). In a recent report from a hospital in Zhejiang, ST463 CRPA emerged and has become the predominant CRPA clone among the population (Hu et al., 2021a). To date, there is still a lack of clinical studies on bloodstream infections of the new-onset KPC-producing CRPA belonging to ST463. Despite the retrospective and single-center nature of this study, it provides a uniquely detailed clinical and microbiological description of a KPC–producing ST463 CRPA BSI cohort from a Chinese hospital.

Conventionally, ST175, ST111, ST235, and ST395 are high-risk PA clones in clinics worldwide (Oliver et al., 2015). However, in our recent two-year study on the CRPA BSI cohort (2019-2020), none of the above international “high-risk” STs were isolated and ST463 has become the dominant CRPA clone in the population causing BSI (48.0%). Baseline characteristics showed no statistical differences in clinical variables across STs. However, 28-day mortality was significantly higher in ST463 cases than in non-ST463 cases. Of the 24 ST463 CRPA BSI cases in the cohort, 16 patients succumbed within 28 days. These BSIs were mainly from the respiratory tract. Indeed, several studies demonstrated that the initial site of infection affects prognosis, with surgery and pneumonia being associated with a particularly poor prognosis for *P. aeruginosa* bloodstream infections (Chatzinikolaou et al., 2000; Feng et al., 2017). In our study, *bla*_KPC_ carriage, Pitt bacteremia score, and underlying hematological disease were three independent risk factors contributing to 28-day fatal outcomes. The survival curves also showed lower survival in KPC-positive ST463 CRPA-infected patients compared to those who are KPC-negative non-ST463 CRPA-infected (**Figures 2D, E**). The detection rate of *bla*_KPC_ was also significantly higher in the ST463 group than in the non-ST463 group (95.8% *vs* 3.8%, *P* < 0.001). This suggests that the dominant clone of CRPA BSIs, ST463, has a high correlation with the *bla*_KPC_ gene and the poor prognosis of BSIs.

Although metallo-*β*-lactamases (MBLs) were found to be the most frequent carbapenemases in CRPA, no MBL genes were found in either ST463 or non-ST463 isolates in this study. Moreover, the *β*-lactamase KPC was produced in almost all ST463 CRPA isolates. This is quite different from the results of our multicenter study conducted in China 10 years ago, when MBL genes including IMP and VIM were detected in 8.5% of CRPA isolates, while no KPC isolates were detected (Wang et al., 2010). Generally, KPC is detected from the *Enterobacteriaceae* family, including *K. pneumoniae* and *E. coli*; this leads to high levels of resistance to carbapenems and other *β*-lactam antibiotics. Therefore, we compared the surrounding sequences of *bla*_KPC_-containing plasmid sequences from different species including *P. aeruginosa* and *Enterobacteriaceae*. The results showed that each plasmid from various species presented core *bla*_KPC-2_ was franked by mobile genetic element IS*Kpn27* and IS*Kpn6* (**Supplementary Figure S1B**). This supports the hypothesis that *bla*_KPC_ in *P. aeruginosa* might be acquired from *Enterobacteriaceae* (Queenan and Bush, 2007; Naas et al., 2013; Hu et al., 2021a).

All 24 ST463 (O4) CRPA isolates carried the *exoU* and *exoS* virulence genes, but not *PldA*, which was somewhat different from the reported strains from 2009 to 2018 (Hu et al., 2021a). Previously *exoU* and *exoS* were always reported to be mutually exclusive (Vareechon et al., 2017), while the coexistence of *exoU* and *exoS* may enhance the resistance of *P. aeruginosa* (Horna et al., 2019). In this study, all ST463 strains carried both *exoS* and *exoU* genes, while non-ST463 strains carried only one of them (15.4% *exoU* and 84.6% *exoS*), consistent with the results of higher resistance of ST463. On the other hand, the group of KPC-ST463-infected larvae had a higher mortality rate than other STs group. In 20% human serum, the maximum growth rate of KPC-positive CRPA was significantly lower compared to KPC-negative CRPA group. Thus, *in vitro* virulence phenotyping experiments showed that KPC was another independent risk factor associated with high lethality. We could further indicate that *exoU* and *exoS* virulence genes coexisting with *bla*_KPC_ resistance gene in ST463 CRPA may be an important intrinsic cause of poor prognosis of clinical PA BSIs. This may lead to a new threat at the clinic.

In 2021, the Infectious Diseases Society of America (IDSA) recommended cefolozane-tazobactam, cefazidime-avibactam, and imipenem-cilastatin-relebactam as monotherapy first-line treatment options for difficult-to-treat *P. aeruginosa* (DTR-PA) infections (Tamma et al., 2021). In our study, the results of antimicrobial susceptibility testing showed that ST463 CRPA isolates were fairly susceptible to amikacin (87.5%) and ceftazidime-avibactam (91.7%). It is suggested that ceftazidime-avibactam or ceftazidime-avibactam combined with amikacin may be a good option for the treatment of infections caused by KPC-producing ST463 CRPA. For adult patients with severe infections, assuming normal renal and liver function, the recommended intravenous dose of ceftazidime-avibactam is 2.5 g every 8 hours, infused over 3 hours. However, more fluoroquinolone-resistant ST463 strains were detected compared to non-ST463 strains, as the prevalence of the *crpP* gene were also significantly higher in ST463 strains (mostly two copies of *crpP*). While no plasmid-mediated coding genes (e.g. *qnr, aac (6’)-Ib-cr*) were found, no other mechanisms of fluoroquinolone resistance such as chromosomal mutation in the DNA gyrase and topoisomerase II/IV-encoding genes (*parC*, *parE*, *gyrA*, and *gyrB*) were further investigated. Another noteworthy issuer, is that most MICs of polymyxin were 1 μg/mL and the effective therapeutic dose should be large and in combination (Nang et al., 2021). For patients on polymyxin B, the recommended loading dose is 2.0- 2.5 mg/kg based on total body weight (TBW) (equivalent to 20,000–25,000 IU/kg) over 1 hour. And the daily maintenance dose is 1.25- 1.5 mg/kg (equivalent to 12,500-15,000 IU/kg TBW) every 12 hours infused over 1 hour (Tsuji et al., 2019). High dose exposure to polymyxin has the potential to cause nephrotoxicity, hence limiting the therapeutic value (Lakota et al., 2018).

In conclusion, we described a BSI cohort by a novel high-risk KPC-PA ST463 clone that has been rapidly emerging and disseminating in East China in recent years. In the current study, we found a higher mortality rate in the ST463 CRPA BSI cohort than in the non-ST463 CRPA BSI. ST463 was the predominant epidemic type of KPC-producing CRPA. The independent risk factors for a 28-day outcome of CRPA BSI were *bla*_KPC_-carrying *P. aeruginosa* and hematological disease. Prompt and effective treatment of ST463 CRPA infection might reduce mortality, especially in patients with underlying hematological disease. These results also suggest that epidemiological surveillance is still needed to monitor the dynamic changes of high-risk clones, such as ST463 CRPA.

## Data Availability Statement

The datasets presented in this study can be found in online repositories. The names of the repository/repositories and accession number(s) can be found in the article/**Supplementary Material**.

## Author Contributions

JW, YZ, MY, QYu, and HF collected clinical data. WS, HH, YC, YZ, HC, and PZ performed laboratory work. YJ, HH, and PZ performed whole-genome sequencing and bioinformatics analysis. TQ, HH, SZ, HF, and PZ analyzed clinical and microbiological data. TQ, HH, and QYa prepared the manuscript. All authors contributed to the article and approved the submitted version.

## Funding

This work was supported by research grants from the National Natural Science Foundation of China (No. NSFC81871689).

## Conflict of Interest

The authors declare that the research was conducted in the absence of any commercial or financial relationships that could be construed as a potential conflict of interest.

## Publisher’s Note

All claims expressed in this article are solely those of the authors and do not necessarily represent those of their affiliated organizations, or those of the publisher, the editors and the reviewers. Any product that may be evaluated in this article, or claim that may be made by its manufacturer, is not guaranteed or endorsed by the publisher.
