# Supplementary material for: Bloodstream Infections Caused by Klebsiella pneumoniae Carbapenemase–Producing P. aeruginosa Sequence Type 463, Associated With High Mortality Rates in China: A Retrospective Cohort Study
Source: Front Cell Infect Microbiol. 2021 Nov 1;11:756782. doi: 10.3389/fcimb.2021.756782 (PMC8592259; doi:10.3389/fcimb.2021.756782)
Supplement: Supplementary file 1 [file DataSheet_1.docx]

**SUPPLEMENTARY METERIALS**

**Supplementary Methods**

1. **Clinical Data Collection**

Data were extracted from the medical records using a standardized data collection form. The following criteria were established before data collection. i) Bacteremia onset was referred to the collection date of the first blood culture yielding the study organism. The source of bacteremia was determined according to the Centers for Disease Control and Prevention definitions (CDC/NHSN 2016 surveillance definitions for specific types of infections. CDC, Atlanta, USA)(1); ii) Nosocomial infections were defined as patients who developed infection after being admitted for more than 48 hours, or the infections were confirmed from another hospital; iii) Pitt bacteremia score is based on five variables: temperature, blood pressure, mechanical ventilation, cardiac arrest, and mental status(2); iv) Prior hospitalization were defined as another hospitalization with two weeks before admission; v)Treatment listed in our study were collected from the date of the first blood culture yielding the study organism. Forty-five patients were included in our clinical study. The variables collected included patients’ demographic data (age, sex and Body Mass Index), underlying diseases, source of bacteraemia, factors such as hospital stay length prior to BSI, previous trauma, surgery, ICU admission prior to bacteremia onset, CRPA BSI prior hemodialysis, and previous invasive procedure, BSI clinical presentations, laboratory examination (such as white blood cell, c reactive protein, albumin and procalcitonin), treatments as the antibiotic dosing regimens, hormonal therapy and other immunotherapy and clinical outcomes (mortality) data at 7, 14 and 28 days.

**2. WGS and bioinformatics analysis**

Whole DNAs of 50 CPRA isolates in our study were extracted using a QIAamp DNA MiniKit (Qiagen, Valencia, CA, USA), and submitted to next-generation high-throughput sequencing (NGS) on the HiSeq2000TM platform (Illumina, San Diego, USA) with 2*100-bp paired-end libraries. Following the quality trimming steps (Phred quality score >20), de novo assembly of the short-read data was carried out on CLC Genomics Workbench version 10.0 (QIAGEN, <https://www.qiagen.com).> Multi-locus sequence typing (MLST) and antibiotic resistance genes were conducted by the CGE server (https://cge.cbs.dtu.dk). To determine the clonal relatedness, we performed a genome-wide gene-by-gene comparison by applying the SeqSphere+ software, v6.0.0 (Ridom GmbH, Münster, Germany), and further analyzed single nucleotide polymorphisms (SNPs) of CRPA isolates of the same Sequence Type clone using Snippy v.4.4.5 (https://github.com/tseemann/snippy) and snp-dists 0.6.3 (https://github.com/tseemann/snp-dists), with default parameters. Isolate ZYPA01 was performed on long-read high-throughput sequencing (LRS) on the MinION platform (Nanopore, Oxford, UK). The NGS and LRS data of both isolates were hybrid assembled by using Unicycler 0.4.8 to obtain the whole genome and particularly complete plasmid sequences. The plasmid was annotated by Prokka (version1.14.5)(3), combined with BLAST searches ([www.ncbi.nlm.nih.gov/blast/](http://www.ncbi.nlm.nih.gov/blast/)). Genome alignments were conducted with Easyfig 2.2.5. Gene organization diagrams were drawn in the CGView Server.

Nucleotide sequence accession number: The complete nucleotide sequence of pZYPA01 was submitted to GenBank under accession number MZ050803.

**3. *Galleria Mellonella* Infection Model**

In order to assess the degree of pathogenicity of the isolates studied, larvae were infected by representative isolates of 5 ST463 CRPA (ZYPA06, ZYPA10, ZYPA29, ZYPA32 and ZYPA36）and 5 non-ST463 CRPA (ZYPA08, ZYPA11, ZYPA23, ZYPA27 and ZYPA41). Overnight bacterial cultures were diluted 1:10 in fresh LB broth and incubated at 37 °C for 4h. The bacteria were diluted using phosphate-buffered saline (PBS) to low, medium and high concentrations (10^5^, 10^6^, 10^7^ CFU/mL). 10μl bacteria inoculum were injected into the first pair of gastropods on the left using 20ul syringes as described previously. Ten larvae weighing between 200 and 250mg were used for each concentration and control larvae were injected with PBS. After injection, G. mellonella were incubated at 37 °C in the dark and observed after 12 h, 24 h, 36 h, 48 h and 72h. Larvae were considered dead when they do not respond to touch and turned black(4). Kaplan Meier analysis with Log Rank test was used for survival analysis.

**4. Serum Resistance Assay**

The assay was tested using LB broth mixed with 20% human serum for a 15-hour incubation as previously reported with some modifications(2). 1μL of overnight culture strain were added into 200μL blank LB broth and LB broth with 20% human serum using a flat-bottom 96-wellplate, as the control and experimental group (n = 3 for each clone). Microplate reader (BioTek, USA) was used to meature absorbance at 600 nm per 5 minutes. The isolates growth curve was drawn.

**Reference**

1. Andrey DO, Pereira Dantas P, Martins WBS, Marques De Carvalho F, Almeida LGP, Sands K, et al. An Emerging Clone, Klebsiellapneumoniae Carbapenemase 2-Producing K. pneumoniae Sequence Type 16, Associated With High Mortality Rates in a CC258-Endemic Setting. Clinical infectious diseases: an official publication of the Infectious Diseases Society of America2020. p. e141-e50.

2. Chow JW, Yu VL. Combination antibiotic therapy versus monotherapy for gram-negative bacteraemia: a commentary. Int J Antimicrob Agents. 1999;11(1):7-12.

3. Seemann T. Prokka: rapid prokaryotic genome annotation. Bioinformatics. 2014;30(14):2068-9.

4. Chen T, Xu Y, Xu W, Liao W, Xu C, Zhang X, et al. Hypertonic glucose inhibits growth and attenuates virulence factors of multidrug-resistant Pseudomonas aeruginosa. BMC Microbiol. 2020;20(1):203.

| **Supplementary Table S1.** Description of the 24 Patients Diagnosed With Bloodstream Infections Caused by CRPA ST463 | | | | | | | | | | | |
| --- | --- | --- | --- | --- | --- | --- | --- | --- | --- | --- | --- |
| Isolate number | Age (sex) | Underlying Disease | Month/Year of Infection | Ward | KPC gene | Source of Bacteriemia | Treatment | Other treatment measures | Outcome at 7 d | Outcome at 28 d | Time From Bacteriemia Onset to Death (d) |
| ZYPA01 | 54 (F) | AML | 9/2020 | Hematology ward (Bone Marrow Transplant Centre) | KPC-2 | UTI | ACF+ATM+PBM | N | died | died | 5 |
| ZYPA06 | 44 (M) | MM | 5/2019 | Hematology ward | KPC-2 | GIT | PMB | N | died | died | 1 |
| ZYPA07 | 26 (F) | ALL | 7/2019 | Hematology ward | KPC-2 | Unknown | IPM+PMB+AMK | N | died | died | 2 |
| ZYPA10 | 66 (M) | Cardiovascular and cerebrovascular disease | 7/2019 | ICU | KPC-2 | deep venous catheter | TZP | Removal of deep venous catheter | alive | alive | alive |
| ZYPA12 | 59 (F) | Diabetes, Pituitary adenoma | 3/2019 | Neurosurgical ward | KPC-2 | Unknown | MEM+AMK | N | alive | alive | alive |
| ZYPA15 | 37 (M) | Chronic liver disease | 2/2020 | ICU | KPC-2 | UTI | PMB+AMK | N | alive | alive | alive |
| ZYPA16 | 40 (F) | Nought | 5/2020 | Orthopedic ward | N | SSI | PMB+AMK | N | alive | alive | alive |
| ZYPA18 | 16 (F) | ALL | 4/2019 | Hematology ward (Bone Marrow Transplant Centre) | KPC-2 | Unknown | MEM+PBM+AMK | N | alive | died | 24 |
| ZYPA19 | 2 (M) | Liver transplantation, Biliary atresia | 11/2020 | Hepatopancreatobiliary Surgery Department | KPC-2 | Lungs | MEM | N | died | died | 7 |
| ZYPA24 | 52 (F) | MM, Cardiovascular and cerebrovascular disease | 5/2019 | ICU | KPC-2 | Unknown | SCF | N | died | died | 1 |
| ZYPA28 | 75 (M) | Cardiovascular and cerebrovascular disease, Diabetes, Chronic liver disease, chronic kidney disease | 11/2020 | Urological ward | KPC-2 | UTI | IPM | Double J tube was placed for drainage | alive | alive | alive |
| ZYPA29 | 22 (M) | Acute promyelocytic leukemia | 10/2020 | ICU | KPC-2 | Unknown | IPM | N | died | died | 0 |
| ZYPA30 | 39 (M) | Liver transplantation | 10/2020 | Hepatopancreatobiliary Surgery Department | KPC-2 | Unknown | PMB | Using immunoglobulin | alive | alive | alive |
| ZYPA31 | 66 (M) | Cardiovascular and cerebrovascular disease | 10/2019 | ICU | KPC-2 | Lungs | MEM+PMB | N | died | died | 3 |
| ZYPA32 | 73 (M) | Cardiovascular and cerebrovascular disease | 2/2019 | ICU | KPC-2 | deep venous catheter | LEV | Removal of deep venous catheter | alive | alive | alive |
| ZYPA34 | 76 (M) | Acute monocytic leukemia | 6/2019 | Hematology ward | KPC-2 | Unknown | PMB | N | died | died | 2 |
| ZYPA36 | 69 (M) | Lung transplantation, COPD, Chronic kidney disease | 1/2019 | ICU | KPC-2 | Lungs | MEM+AMK | N | died | died | 5 |
| ZYPA37 | 53 (M) | Severe pneumonia, Chronic liver disease | 12/2019 | ICU | KPC-2 | Lungs | IPM | N | died | died | 1 |
| ZYPA38 | 25 (M) | ALL, Diabetes | 8/2020 | Hematology ward (Bone Marrow Transplant Centre) | KPC-2 | Unknown | ACF+PMB | N | alive | alive | alive |
| ZYPA39 | 43 (M) | Acute monocytic leukemia | 6/2019 | Hematology ward | KPC-2 | Unknown | IPM+PMB | N | died | died | 3 |
| ZYPA46 | 72 (M) | Cardiovascular and cerebrovascular disease, Mantle cell lymphoma | 10/2019 | ICU | KPC-2 | Unknown | MEM | N | died | died | 1 |
| ZYPA48 | 49 (M) | NHL, Chronic liver disease | 9/2019 | Hematology ward | KPC-2 | Unknown | MEM | N | died | died | 7 |
| ZYPA52 | 19 (M) | ALL | 5/2019 | Hematology ward | KPC-2 | Unknown | MEM+AMK | N | died | died | 7 |
| ZYPA54 | 25 (M) | N | 10/2019 | ICU | KPC-33 | Lungs | ACF+PMB+AMK | N | alive | died | 13 |

Abbreviations: N: none; AML, acute myeloid leukemia; ALL, acute lymphoblastic leukemia; MM: Multiple myeloma; NHL, non-Hodgkin lymphoma; UTI, urinary tract infections; SSI, surgical site infections; GIT, gastrointestinal tract; ICU, intensive care unit; ATM, aztreonam; PMB, polymyxin B; BIM, biapenem; IPM, imipenem; MEM, meropenem; AMK, amikacin; SCF, cefoperazone-sulbactam; TZP, piperacillin-tazobactam; LEV, levofloxacin; ACF, Ceftazidime-Avibatanm.

Notably, 10 cases (41.7%) were from the ICU, followed by 9 cases (37.5%) from the hematology ward (including 3 cases from the bone marrow transplantation center), and 5 cases (20.8%) in the surgical ward. The patients had a wide age distribution (2 to 76 years) with a male to female ratio of 18:6. Most patients had underlying hematologic disease (n=12, 50.0%), some cardiovascular disease (n=6), hepatobiliary disease (n=6), and solid-organ transplantation (n=2). Bacteremia was mainly from respiratory tract infections (6 cases) and urinary tract infections (3 cases). The treatment regimen of patients after obtaining positive blood culture specimens was documented: carbapenem combined with amikacin was the most common combination therapy prescribed (n=5, 20.8%); the combination of polymyxin and carbapenem accounted for 16.7% (n=4); the combination of ceftazidime-avibactam and polymyxin accounted for 12.5% (n=3); the combination of ceftazidime-avibactam and amikacin accounted for 4.2% (n=1). 6 patients did not receive adjusted anti-infective therapy due to premature death. 67.0% (n=16) patients died within 28 days, of which 58.3% (n=14) died within 7 days.

**Supplementary Table S2.** The Genomic Sequence Datasets

| Numbers | Object IDs | corresponding URLs |
| --- | --- | --- |
| ZYPA01 | JAIQLG000000000 | https //www.ncbi.nlm.nih.gov/wgs_batch/JAIQLG000000000 |
| ZYPA02 | JAIQLF000000000 | https //www.ncbi.nlm.nih.gov/wgs_batch/JAIQLF000000000 |
| ZYPA04 | JAIQLE000000000 | https //www.ncbi.nlm.nih.gov/wgs_batch/JAIQLE000000000 |
| ZYPA06 | JAIQLD000000000 | https //www.ncbi.nlm.nih.gov/wgs_batch/JAIQLD000000000 |
| ZYPA07 | JAIQLC000000000 | https //www.ncbi.nlm.nih.gov/wgs_batch/JAIQLC000000000 |
| ZYPA08 | JAIQLB000000000 | https //www.ncbi.nlm.nih.gov/wgs_batch/JAIQLB000000000 |
| ZYPA09 | JAIQLA000000000 | https //www.ncbi.nlm.nih.gov/wgs_batch/JAIQLA000000000 |
| ZYPA10 | JAIQKZ000000000 | https //www.ncbi.nlm.nih.gov/wgs_batch/JAIQKZ000000000 |
| ZYPA11 | JAIQKY000000000 | https //www.ncbi.nlm.nih.gov/wgs_batch/JAIQKY000000000 |
| ZYPA12 | JAIQKX000000000 | https //www.ncbi.nlm.nih.gov/wgs_batch/JAIQKX000000000 |
| ZYPA13 | JAIQKW000000000 | https //www.ncbi.nlm.nih.gov/wgs_batch/JAIQKW000000000 |
| ZYPA14 | JAIQKV000000000 | https //www.ncbi.nlm.nih.gov/wgs_batch/JAIQKV000000000 |
| ZYPA15 | JAIQKU000000000 | https //www.ncbi.nlm.nih.gov/wgs_batch/JAIQKU000000000 |
| ZYPA16 | JAIQKT000000000 | https //www.ncbi.nlm.nih.gov/wgs_batch/JAIQKT000000000 |
| ZYPA17 | JAIQKS000000000 | https //www.ncbi.nlm.nih.gov/wgs_batch/JAIQKS000000000 |
| ZYPA18 | JAIQKR000000000 | https //www.ncbi.nlm.nih.gov/wgs_batch/JAIQKR000000000 |
| ZYPA19 | JAIQKQ000000000 | https //www.ncbi.nlm.nih.gov/wgs_batch/JAIQKQ000000000 |
| ZYPA20 | JAIQKP000000000 | https //www.ncbi.nlm.nih.gov/wgs_batch/JAIQKP000000000 |
| ZYPA21 | JAIQKO000000000 | https //www.ncbi.nlm.nih.gov/wgs_batch/JAIQKO000000000 |
| ZYPA22 | JAIQKN000000000 | https //www.ncbi.nlm.nih.gov/wgs_batch/JAIQKN000000000 |
| ZYPA23 | JAIQKM000000000 | https //www.ncbi.nlm.nih.gov/wgs_batch/JAIQKM000000000 |
| ZYPA24 | JAIQKL000000000 | https //www.ncbi.nlm.nih.gov/wgs_batch/JAIQKL000000000 |
| ZYPA25 | JAIQKK000000000 | https //www.ncbi.nlm.nih.gov/wgs_batch/JAIQKK000000000 |
| ZYPA26 | JAIQKJ000000000 | https //www.ncbi.nlm.nih.gov/wgs_batch/JAIQKJ000000000 |
| ZYPA27 | JAIQKI000000000 | https //www.ncbi.nlm.nih.gov/wgs_batch/JAIQKI000000000 |
| ZYPA28 | JAIQKH000000000 | https //www.ncbi.nlm.nih.gov/wgs_batch/JAIQKH000000000 |
| ZYPA29 | JAIQKG000000000 | https //www.ncbi.nlm.nih.gov/wgs_batch/JAIQKG000000000 |
| ZYPA30 | JAIQKF000000000 | https //www.ncbi.nlm.nih.gov/wgs_batch/JAIQKF000000000 |
| ZYPA31 | JAIQKE000000000 | https //www.ncbi.nlm.nih.gov/wgs_batch/JAIQKE000000000 |
| ZYPA32 | JAIQKD000000000 | https //www.ncbi.nlm.nih.gov/wgs_batch/JAIQKD000000000 |
| ZYPA33 | JAIQKC000000000 | https //www.ncbi.nlm.nih.gov/wgs_batch/JAIQKC000000000 |
| ZYPA34 | JAIQKB000000000 | https //www.ncbi.nlm.nih.gov/wgs_batch/JAIQKB000000000 |
| ZYPA35 | JAIQKA000000000 | https //www.ncbi.nlm.nih.gov/wgs_batch/JAIQKA000000000 |
| ZYPA36 | JAIQJZ000000000 | https //www.ncbi.nlm.nih.gov/wgs_batch/JAIQJZ000000000 |
| ZYPA37 | JAIQJY000000000 | https //www.ncbi.nlm.nih.gov/wgs_batch/JAIQJY000000000 |
| ZYPA38 | JAIQJX000000000 | https //www.ncbi.nlm.nih.gov/wgs_batch/JAIQJX000000000 |
| ZYPA39 | JAIQJW000000000 | https //www.ncbi.nlm.nih.gov/wgs_batch/JAIQJW000000000 |
| ZYPA40 | JAIQJV000000000 | https //www.ncbi.nlm.nih.gov/wgs_batch/JAIQJV000000000 |
| ZYPA41 | JAIQJU000000000 | https //www.ncbi.nlm.nih.gov/wgs_batch/JAIQJU000000000 |
| ZYPA42 | JAIQJT000000000 | https //www.ncbi.nlm.nih.gov/wgs_batch/JAIQJT000000000 |
| ZYPA43 | JAIQJS000000000 | https //www.ncbi.nlm.nih.gov/wgs_batch/JAIQJS000000000 |
| ZYPA44 | JAIQJR000000000 | https //www.ncbi.nlm.nih.gov/wgs_batch/JAIQJR000000000 |
| ZYPA45 | JAIQJQ000000000 | https //www.ncbi.nlm.nih.gov/wgs_batch/JAIQJQ000000000 |
| ZYPA46 | JAIQJP000000000 | https //www.ncbi.nlm.nih.gov/wgs_batch/JAIQJP000000000 |
| ZYPA47 | JAIQJO000000000 | https //www.ncbi.nlm.nih.gov/wgs_batch/JAIQJO000000000 |
| ZYPA48 | JAIQJN000000000 | https //www.ncbi.nlm.nih.gov/wgs_batch/JAIQJN000000000 |
| ZYPA49 | JAIQJM000000000 | https //www.ncbi.nlm.nih.gov/wgs_batch/JAIQJM000000000 |
| ZYPA50 | JAIQJL000000000 | https //www.ncbi.nlm.nih.gov/wgs_batch/JAIQJL000000000 |
| ZYPA52 | JAIQJK000000000 | https //www.ncbi.nlm.nih.gov/wgs_batch/JAIQJK000000000 |
| ZYPA53 | JAIQJJ000000000 | https //www.ncbi.nlm.nih.gov/wgs_batch/JAIQJJ000000000 |
| ZYPA54 | JAIQJI000000000 | https //www.ncbi.nlm.nih.gov/wgs_batch/JAIQJI000000000 |


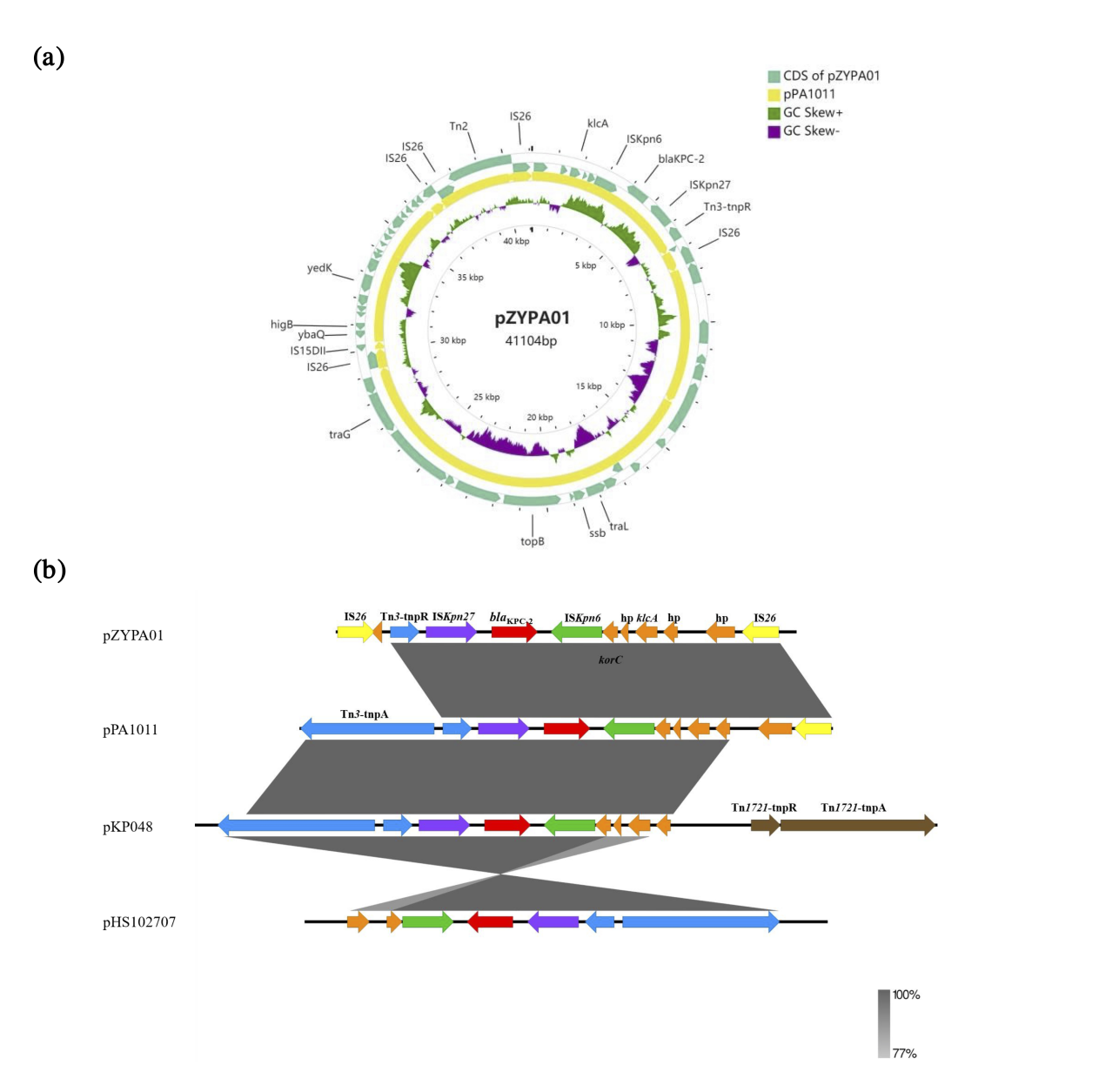


**Supplementary Figure S1. a)** Genetic organization of pZYPA01 and comparison with similar plasmid pPA1011 (GenBank accession number MH734334.1) from *P. aeruginosa*. It harbored multiple resistance genes, including one aminoglycoside resistance gene (ant(2’’)-Ia), one sulphonamide resistance gene (sul1) and two β-lactam resistance genes (blaKPC-2, blaCARB-2). The ZYPA01 isolate contained only one loop-closed DNA sequence (a single plasmid), designated pZYPA01, which was 41104 bp in size with an average G+C content of 58.4%, encoding 57 predicted open reading frames (ORFs) including five copies of IS26. **b)** *bla*_KPC-2_-harboring genetic elements in pZYPA01 (a plasmid from an isolate in our study ZYPA01, GenBank accession number, MZ050803), in pPA1011 (a plasmid from a ST463 *P. aeruginosa* isolate in Hangzhou, China), in pKP048 (plasmid from *K. pneumoniae*) and in pHS102707 (plasmid from *Escherichia coli*). Shading indicates regions of homology.
